# In-situ measurements of wall moisture in a historic building in response to the installation of an impermeable floor

**DOI:** 10.14324/111.444/ucloe.000046

**Published:** 2022-11-08

**Authors:** Kevin Briggs, Richard Ball, Iain McCaig

**Affiliations:** 1 Department of Architecture and Civil Engineering, University of Bath, Bath, BA2 7AY, UK; 2 Historic England, Swindon, UK

**Keywords:** masonry, wall moisture, historic building, conservation, renovation, capillary rise, evaporation, timber dowel, soil moisture deficit

## Abstract

When impermeable ground bearing slabs are installed in old buildings without a damp-proof course, it is a common belief of conservation practitioners that ground moisture will be ‘driven’ up adjacent walls by capillary action. However, there is limited evidence to test this hypothesis.

An experiment was used to determine if the installation of a vapour-proof barrier above a flagstone floor in a historic building would increase moisture content levels in an adjacent stone rubble wall. This was achieved by undertaking measurements of wall, soil and atmospheric moisture content over a 3-year period. Measurements taken using timber dowels showed that the moisture content within the wall did not vary in response to wall evaporation rates and did not increase following the installation of a vapour-proof barrier above the floor. This indicates that the moisture levels in the rubble wall were not influenced by changes in the vapour-permeability of the floor.

## Introduction

Water movement through the masonry walls of historic buildings is an important process influencing thermal performance, wall deterioration (e.g., salt weathering), decay of built-in timbers, and damage to the internal finishes and environment (e.g., mould) [1]. Therefore, understanding moisture regimes within historic structures is critical to heritage conservation and the appropriate selection of materials for repair or renovation [2].

Relatively impermeable concrete ground-bearing slabs are sometimes installed in historic buildings during renovation, but it is unclear if this adversely alters the moisture dynamics of the building. It is believed by many conservation practitioners that if an impermeable ground bearing slab is installed in a historic building during renovation, and particularly those which do not contain a damp-proof course, ground moisture will be ‘driven’ up adjacent walls through capillary action. Although there are references to this phenomenon in the technical literature [3,4], there is limited evidence based on long-term monitoring.

**Figure 1:**
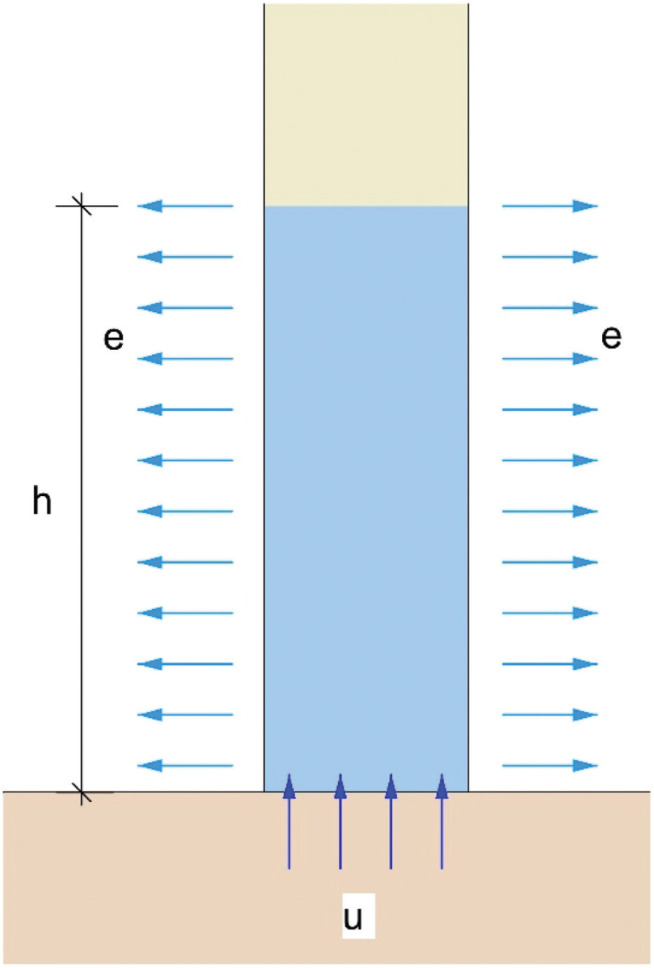
A conceptual model of wall damp rise by Hall and Hoff [7], with soil moisture uptake (u) and wall evaporation (e) driving the saturated wall height (h), in a wall thickness (b).

Water transport in buildings and building materials is site-specific and complex, but is generally dominated by capillary forces and unsaturated flow within the pores of building materials [2,5,6]. It can be difficult to model unsaturated flow through historic masonry walls because it is not always possible to intrusively characterise the physical properties of the wall materials, their heterogeneity and the interfaces between the different materials forming the wall. However, it is possible to measure and model the primary processes influencing the supply and removal of water within walls, to consider the behaviour of the wall as a system. Hall and Hoff [7] developed a quantitative representation of the primary processes controlling moisture migration and wall damp rise in a masonry wall (Fig. 1). This shows that ground moisture (*u*) is absorbed at the base of a homogenous, porous wall of thickness *b*. To ensure the conservation of mass, a state of dynamic equilibrium is established if the height of the wetted part of the wall (*h*) varies in response to the evaporation rate on the wall surface (*e*).

The Hall and Hoff [7] conceptual model describes the primary process of moisture migration through porous building materials. However, the wall moisture dynamics in a historic building is further complicated by in situ conditions that are difficult to quantify, measure and model. This includes but is not limited to (i) the heterogeneous nature of old masonry walls, and (ii) the moisture storage and conductivity properties of the materials forming the wall. Therefore, in situ monitoring was identified as the most effective method to measure the response of a masonry wall to the installation of an impermeable floor barrier in a historic building.

## Aim and objectives

The aim was to determine if moisture levels in the external wall of a historic building were responsive to seasonal potential evaporation rates and if these were influenced by the installation of an impermeable ground bearing slab. The first objective was to determine whether seasonal changes in soil moisture content and evaporative drying influenced moisture levels within the wall. The second objective was to simulate the installation of an impermeable ground bearing slab by sealing the floor with a vapour-proof barrier and measuring changes in wall moisture levels due to the intervention.

## Method

### The monitoring programme

**Figure 2:**
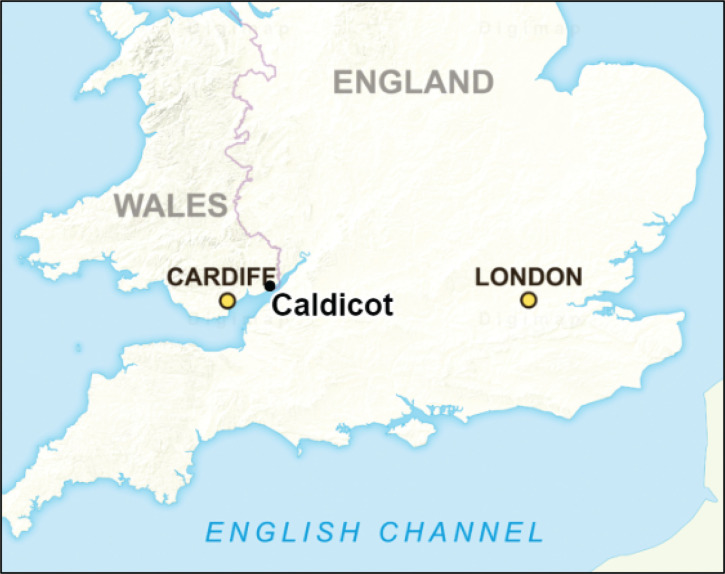
Court House is located in Caldicot, Wales. © Crown copyright and database rights 2021 Ordnance Survey (100025252) using Digimap Ordnance Survey Collection, https://digimap.edina.ac.uk/.

**Figure 3:**
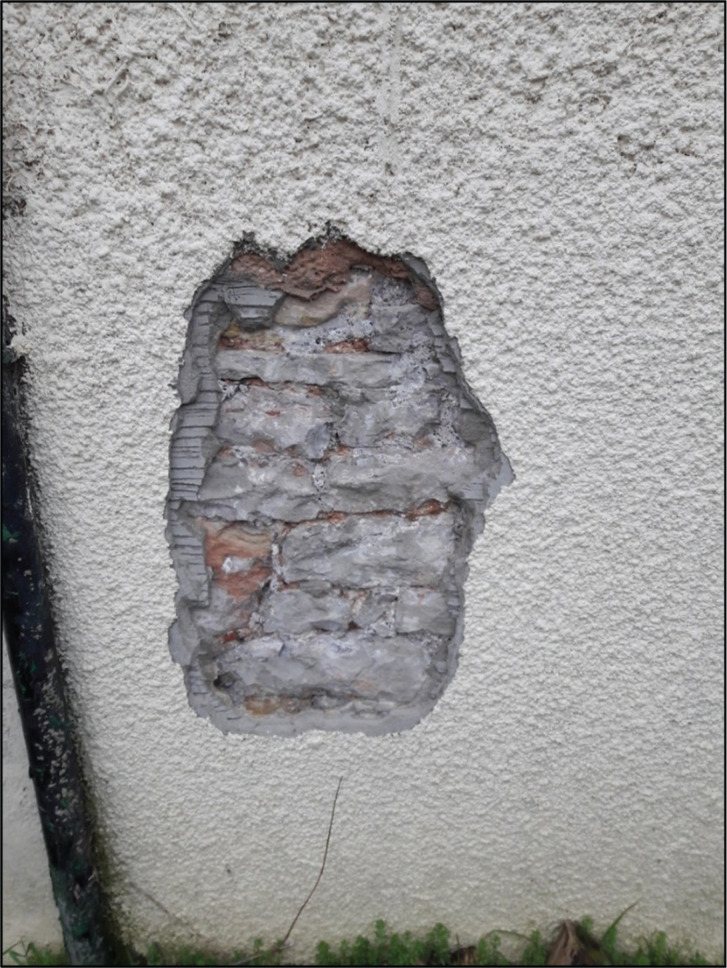
The external face of the pantry wall at Court House.

A 3-year monitoring programme was undertaken to measure moisture levels in a 600 mm thick, composite rubble-core masonry wall at Court House; a private residential property in Caldicot, Wales (Fig. 2). Court House is a Grade II listed building originating from the 16th or 17th Century [8]. The masonry wall was located on the north elevation of the house and formed the external wall of an unheated, flagstone-floored room that was used as a pantry during the monitoring period (Fig. 3).

**Figure 4:**
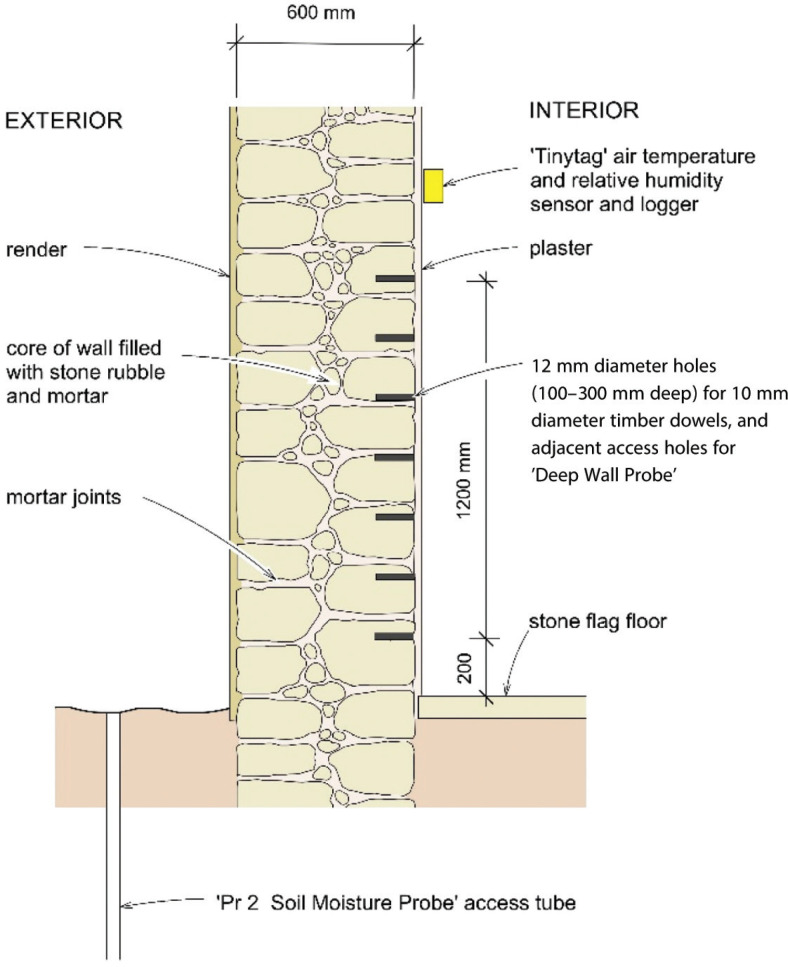
Instrumentation installed in a 600 mm thick, rubble-core masonry wall at Courth House to measure wall moisture (%), soil moisture (%) and evaporative drying on the wall face.

The monitoring programme included measurements of soil moisture levels at the base of the wall, the evaporative drying on the wall faces and the moisture levels within the wall (Fig. 4), in accordance with the conceptual model (Fig. 1). Details of the electronic sensors are shown in Table 1.

**Table 1.** A summary of electronic sensors installed at Court House

Type of instrument	Measurement	Location	Source/references
WS-GP1 weather station	Solar radiation (kWm^‒2^), air temperature (°C), humidity (%), rainfall (mm/tip), wind speed (ms^‒1^), wind direction (°)	Court House (external)	Delta-T Devices, Ltd, Cambridge, UK
Tinytag Plus 2	Internal air temperature (°C) and relative humidity (%)	Internal wall at Court House	Gemini Data Loggers, Chichester, UK
Protimeter Mini, with deep wall probe	Wall moisture ‘wood moisture equivalent’ (via resistance)(%)	Holes drilled into internal wall face at Court House (0.2 m–1.4 m above ground level)	Amphenol Advanced Sensors, Taunton, UK
PR2 Profile Probe	Soil moisture (m^3^m^−3^)	Court House (0 m–1 m below ground level)	Delta-T Devices, Ltd, Cambridge, UK

The internal structure of the wall was unknown, but walls elsewhere in the property were formed from an inner and outer leaf of stone rubble bedded in lime mortar, with a mortar and rubble-filled core. The wall was rough cast cement rendered and painted on the external face (Fig. 3). It was plastered with a lime-based material and painted on the internal face. A shallow excavation at the property showed that the ground consisted of clay soil mixed with made ground and infilled ground [9], overlain by organic topsoil. A geological map showed that Court House is located on an outcrop of sandstone from the Mercia Mudstone Group [10] but this was not observed in the ground excavation.

The instrument installation and monitoring began in January 2017. Site visits to measure soil and wall moisture levels were undertaken at approximately monthly intervals between January 2017 and March 2020. A vapour-proof, 0.15 mm thick polyethylene sheet was laid over the floor along the length of the pantry wall and sealed at the edges with tape on 18th September 2019, to simulate the installation of an impermeable ground bearing slab. Supporting laboratory experiments showed that the polyethylene sheet was less permeable than a concrete slab. It therefore simulated a worst-case scenario in terms of creating an impermeable floor. A Tinytag logger was installed beneath the polyethylene sheet to measure the time required to reach a constant humidity (%) reading.

### Instrumentation and measurement

Figure 4 shows the layout of instrumentation installed at Court House. Evaporative conditions on the internal and external wall faces were continually measured and logged. Externally, a WS-GP1 weather station [11] was installed to measure hourly changes in solar radiation (kWm^‒2^), air temperature (°C), humidity (%), rainfall (mm/tip), wind speed (ms^‒1^) and wind direction (°). A Tinytag Plus 2 datalogger [12] was installed at the top of the internal wall face to measure hourly changes in air temperature (°C) and relative humidity (%) directly adjacent to the wall surface.

Soil moisture was measured using a PR2 Soil Moisture Probe [13,14], inserted into a 1 m long access tube at approximately monthly intervals. The probe has electronic sensors fixed to a 25 mm diameter polycarbonate rod at fixed intervals of 0.1 m, 0.2 m, 0.3 m, 0.4 m, 0.6 m and 1 m below ground level. The sensing elements measure the permittivity (ε) of the soil in a 100 mm radius surrounding the probe. These were logged and converted to volumetric moisture content (*θ*, %) using a linear relationship for mineral soils:

(1) $$\theta =\frac{\sqrt{\varepsilon }-1.6}{8.4}$$

Changes in wall moisture were measured indirectly at approximately monthly intervals using (i) timber dowels and (ii) a commercial moisture meter. Seven, 130 mm long, 12 mm diameter holes were drilled into the internal face of the pantry wall in a vertical array at 0.2 m spacing between 0.2 m and 1.4 m above ground level (Fig. 4). Pine dowels (10 mm diameter) were installed into these holes and sealed with plumber’s putty. Weight measurements of the dowels were then taken on site at approximately monthly intervals, to determine changes in their gravimetric moisture content (%). Calibration of the timber dowels showed that they took approximately 14 days to reach equilibrium and provided a good indicator of relative changes in wall moisture, however, absolute values at dowel moisture contents > 15% may be underestimated.

At approximately monthly intervals, ‘deep wall probes’ were inserted into the wall to measure wall moisture using a Protimeter Mini Moisture Meter [15]. The deep wall probes were inserted into seven pairs of 75 mm deep, 6 mm diameter holes that were drilled 40 mm horizontally apart, directly adjacent to the larger diameter holes which contained the timber dowels, again between 0.2 m and 1.4 m above ground level. These holes were also sealed with plumber’s putty. The Protimeter Mini moisture meter provides a ‘wood moisture equivalent’ reading (6–90%) based on the electrical resistance measured between the probes. The calibration for the Protimeter Mini Moisture Meter was not readily available from the manufacturer, so the meter readings were treated as an approximate measure of relative changes in wall moisture.

### Interpretation of potential evaporative drying

An approximation for the potential evaporative conditions on the external and internal wall faces were calculated from the weather station and Tinytag measurements of air temperature (°C) and relative humidity (%). The potential evaporation was assumed to be equal to the potential evapotranspiration (*PET*) calculated using the simple equation by Schendel [16] and appraised for climate modelling by Bormann [17]:

(2) $$PET=\frac{16\cdot T}{RH}$$

where *PET* is the potential evapotranspiration (mm/day), *T* is the mean daily temperature (°C) and *RH* is the mean daily relative humidity (%).

### Interpretation of soil moisture

The soil moisture levels at the property were calculated from (i) the weather station data and (ii) direct measurements of the soil moisture content profile. Using both approaches it was possible to calculate a soil moisture deficit (SMD) for the soil profile between 0 m and 1 m below ground level.

The SMD is the volume of water per unit area (mm^3^mm^‒2^) that the soil can absorb before reaching field capacity, where the moisture content is in equilibrium and free to drain under gravity [18]. The daily SMD can be calculated from a soil water balance of daily rainfall infiltration and potential evapotranspiration; bounded by SMD equal to zero when the soil is at field capacity and water cannot infiltrate the soil surface. The daily SMD at Court House was calculated using the rainfall, temperature and relative humidity measurements from the weather station, with the PET calculated using Equation 2.

The measured SMD (*SMD_m_*) was derived from the PR2 Profile Probe measurements of volumetric moisture content (*θ*) using the approach described by Smethurst et al. [19]. The total *SMD_m_* of the soil profile (0–1 m below ground level) was calculated using

(3) $$SMD_{m}=\sum_{i}^{n}h_{i}(\theta_{FC}-\theta_{i})$$

where *θ_i_* is the measured volumetric moisture content in each soil layer (*n*), of thickness *h_i_*. A volumetric moisture content of 38% was assumed at field capacity (*θ_FC_*), based on the wettest soil profiles measured.

### Interpretation of wall moisture

Timber dowels have been used to successfully measure in situ moisture changes in solid brick walls [20] and historic stone walls [21]. Timber dowels absorb moisture over 2 or 3 weeks until they achieve equilibrium with the surrounding wall [22]. Prior to installation, the timber dowels were oven dried at 105 °C for at least 24 h to determine the dry mass (*m_d_*). The timber dowels were then weighed at monthly intervals to measure the wet mass (*m_w_*) and enable calculation of the relative changes in gravimetric moisture content (*w_m_*) using:

(4) $$w_{m}(\%)=\frac{\left(m_{w}-m_{d}\right)}{m_{d}}\times 100\%$$

It was possible to calculate the wall moisture changes using the potential evaporative drying measurements, for comparison with the timber dowels measurements. Hall and Hoff [7] derived a conceptual model for rising damp moisture movement within a porous masonry wall without finishes (Fig. 1). From this they developed a one-dimensional model of capillary rise dynamics based on sharp front theory. The model shows that water will rise within the pores of a wall via capillary action, if the wall has interconnected pore space and water is available at the base of the wall. Hall and Hoff [7] showed that the steady-state height of water rise (*h_ss_*) within a porous wall can be calculated using:

(5) $$h_{ss}=S{\left(\frac{b}{2e\theta_{w}}\right)}^{1/2}$$

where *S* is the sorptivity of the masonry (mm.min^‒1/2^), *e* is the evaporation rate (mm.min^‒1^), *θ_w_* is the moisture content of the wetted part of the wall (mm^3^.mm^‒3^) and *b* is the wall thickness (mm). Equation 5 was used to calculate the daily, steady-state height of water rise using the daily average *PET* (mm.min^‒1^) measured at Court House on both the internal and external wall faces. The wall thickness (*b*) was 600 mm. The sorptivity (*S*) and moisture content of the wetted part of the wall (*θ_w_*) were not measured but were assumed to be 1.0 mm.min^‒1/2^ and 0.2, respectively, as used by Hall and Hoff [7].

## Results

### Evaporative drying

**Figure 5:**
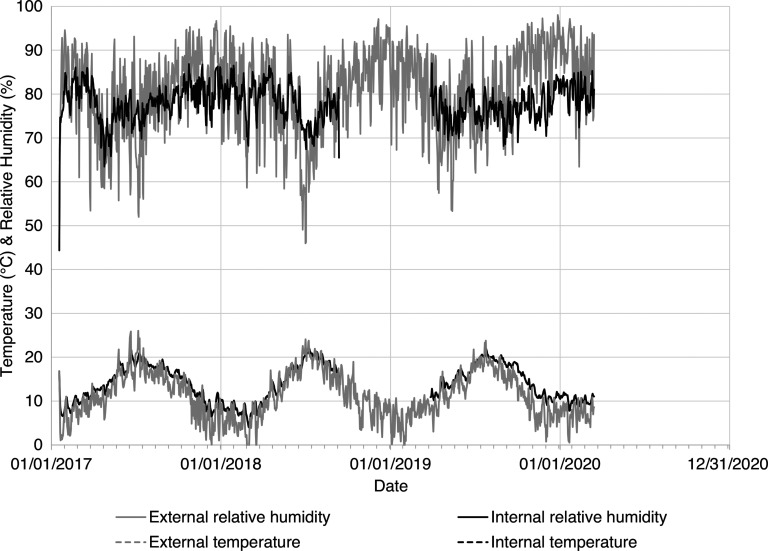
Daily average temperature (*°*C) and relative humidity (%) measured internally (Tinytag logger data) and externally (weather station data) at Court House between 2017 and 2020. Note that internal Tinytag data are missing from September 2018 to March 2019 due to instrument damage.

**Figure 6:**
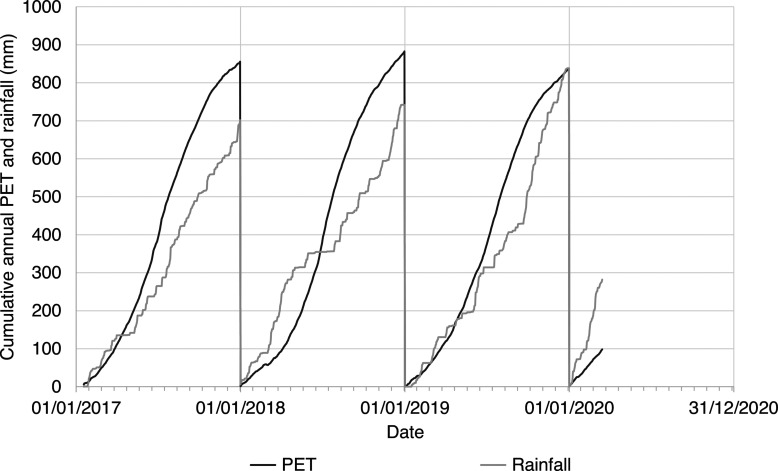
Cumulative annual evapotranspiration (ET0, mm) and rainfall (mm) measured by the weather station at Court House. Note that the measurements start on 18/01/2017.

The internal and external temperature (°C) and relative humidity (%) data showed potential evaporative drying during the summer months, followed by reduced drying through the winter months. These seasonal changes are typical of the temperate UK climate [23,24]. Figure 5 shows increased temperature and reduced relative humidity in the summer months (April to September), relative to the cooler, more humid winter months (October to March). A comparison of annual cumulative potential evapotranspiration (*PET*, mm) and rainfall (mm) shows that *PET* was greatest in the summer months and least in the winter months, with consistent total, annual cumulative *PET* (Fig. 6). The calculated cumulative potential evapotranspiration was higher than comparative measurements in southern England [25,26], due to the simple *PET* model used (Equation 2). Figure 6 shows that 2018 was both wetter (January to June) and drier (July to December) than in 2017 and 2019. According to the conceptual model of wall damp rise (Fig. 1) and Equation 5, these evaporative conditions would lead to greater annual variation in the wall damp rise (mm) in 2018 than in the preceding or succeeding years (2017 and 2019).

### Soil moisture

**Figure 7:**
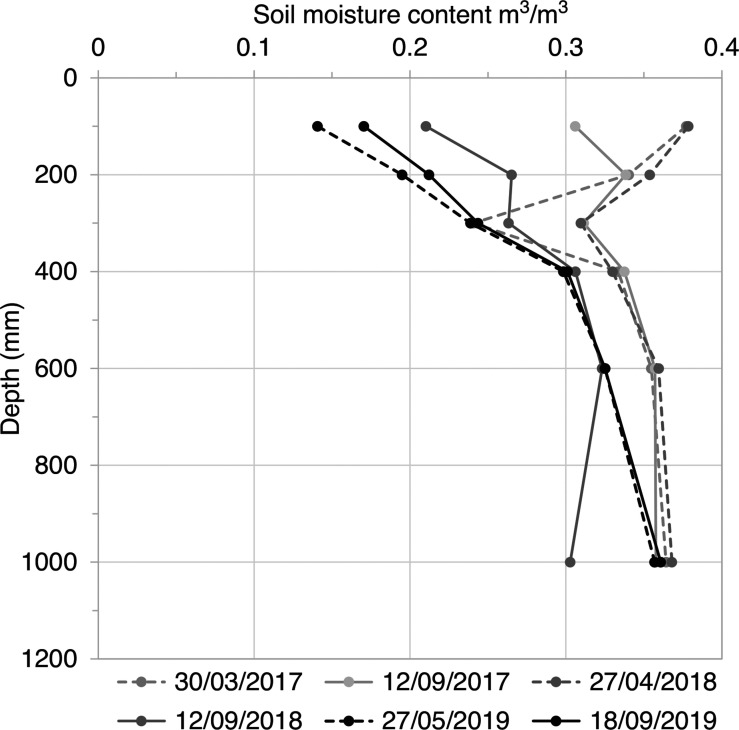
Soil moisture content profiles measured at the end of winter (April/May) and the end of summer (July/September) at Court House.

**Figure 8:**
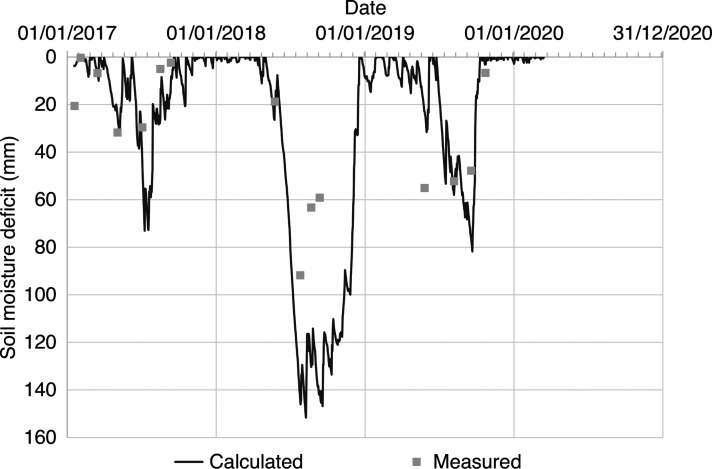
SMD (mm) at Court House (i) calculated using daily weather station data and (ii) measured using a PR2 soil moisture probe (up to 1 m below ground level).

Figure 7 shows soil moisture content profiles measured at the end of winter and the end of summer between 2017 and 2019. The greatest variation in soil moisture content occurred in the near surface, up to 0.4 m below ground level, as is typical in clay soils with grass vegetation at equivalent latitude [18]. Figure 7 shows that the soil moisture content was often below field capacity (*θ_FC_* = 38%) and that a supply of water was not consistently available at the base of the masonry wall. Figure 8 shows that soil moisture was available at the base of the masonry wall during the winter months (i.e., SMD = 0), while there was a soil moisture deficit (i.e., SMD > 0) during the summer months. This shows that the availability of soil moisture varied seasonally and was not constant, as was assumed in the conceptual model (Fig. 1).

### Wall moisture

**Figure 9:**
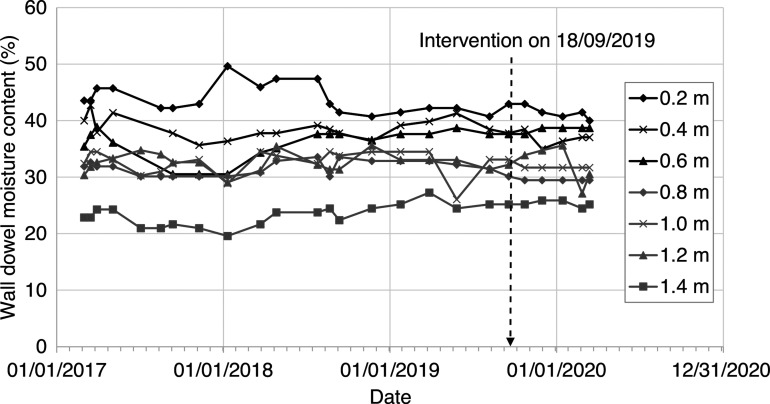
Measurements of timber dowel moisture content (by mass) between 0.2 m and 1.4 m above ground level over a 3-year period between 2017 and 2020, including an intervention to seal the floor on 18/09/2019.

Figure 9 shows the dowel moisture (mass) content values taken at approximately monthly intervals between March 2017 and March 2020. The measurements show that the dowel was close to 50% moisture content at the base of the wall and consistently greater than higher up the wall. The data show that the moisture level of the dowels, and by implication the wall, did not vary in response to seasonal evaporation rates. Nor did the dowel moisture levels immediately increase in response to the sealing of the flagstone floor. Measurements with a Tinytag logger (not shown in Fig. 4) showed that moisture levels rapidly increased beneath the vapour-proof barrier within 2 days of installation in September 2019, showing ground moisture transfer through the floor and into the internal environment of the room.

**Figure 10:**
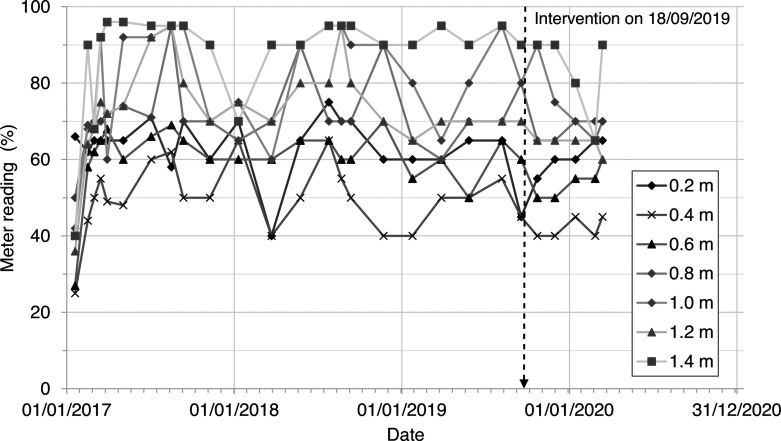
Protimeter Mini Moisture Meter readings measured between 0.2 m and 1.4 m above ground level over a 3-year period between 2017 and 2020, including an intervention to seal the floor on 18/09/2019.

Figure 10 shows the wall moisture levels measured using the moisture meter with a deep wall probe. The wall moisture probe showed consistently lower meter readings at the base of the wall relative to the upper part of the wall. The meter readings were erratic and did not show a temporal trend. It is possible that the meter readings were responding to changes in the internal air temperature and humidity or were influenced by the distribution of salts within the wall [27]. They were not considered to be reliable measurements of wall moisture levels for this study.

**Figure 11:**
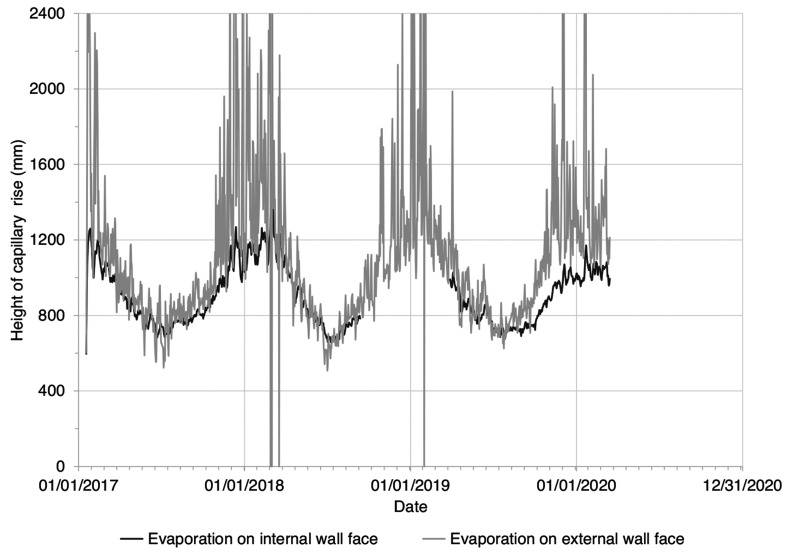
The height of wall capillary rise calculated using the Hall and Hoff [7] sharp front model using PET derived from temperature and relative humidity data measured (i) on the internal wall face and (ii) on the external wall face. Note: Internal wall face data are missing for September 2018 to March 2019. Extreme capillary rise values for the external wall face have been omitted for clarity.

Figure 11 shows the height of wall capillary rise calculated using the Hall and Hoff [7] sharp front model, assuming (i) the supply of water at the base of the wall (ii) potential evaporative drying measured on the internal and external wall faces at Court House and (iii) a porous masonry wall with interconnected pores and without finishes. This shows that prior to the installation of the vapour-proof barrier, given the model assumptions, the capillary rise should have varied between 800 mm (summer) and 1200 mm (winter) above ground level. However, the historic masonry wall was not subject to capillary rise, despite the supply of water at the wall base (Fig. 8) and seasonally variable evaporative drying on the wall face (Fig. 6). Inspection of the wall showed that the wall was formed from porous materials, but with large voids and discontinuities that would inhibit capillary flow. Therefore, the fabric of the wall itself did not facilitate water being ‘driven up’ by capillary action. This was confirmed by the measurements showing that the wall moisture levels did not vary seasonally, nor did they vary in response to the installation of a vapour-proof barrier to seal the floor (Fig. 9).

Pre-existing moisture damage was observed on the internal plaster surface of the lower part of the wall (approx. 200 mm above ground level) prior to instrumentation, but the moisture levels did not vary at this location during the monitoring period. It is possible that localised capillary rise occurred within the plaster and caused the damage. However, this did not affect the core of the wall, nor was the base of the wall influenced by the installation of the vapour-proof barrier during the monitoring period.

## Conclusions

Instrumentation was installed in a historic building to measure changes in wall moisture content and to measure the response of the wall to vapour-sealing of the ground floor. The monitoring programme was based on a conceptual model of capillary rise within the pores of the wall, driven by evaporative drying on the wall surface.

The following conclusions can be drawn from the results presented:

1. The rubble-fill, masonry wall at Court House was not susceptible to wall moisture fluctuations due to capillary rise, driven by evaporative drying. The moisture levels in the wall did not vary in response to changes in potential evaporative drying on the internal and external faces of the wall, despite the availability of soil moisture at the base of the wall during the winter months.
2. Measurements of soil moisture content showed that the supply of water from the soil is seasonally variable. Water is often not available for capillary rise within the pores of a wall during the drier summer months, when soil moisture levels are below field capacity. The supply of water for capillary uptake within a wall is greatest during the winter months, when the ground is more likely to be close to, or at field capacity. This seasonal variation is comparable to measurements in other clay soils in the south of England [18,19].
3. If an impermeable ground bearing slab was installed in this building, ground moisture would not necessarily be ‘driven’ up adjacent walls. Measurements beneath the vapour-proof barrier confirmed that moisture was moving through the flagstone floor, but this did not increase the wall moisture. Sealing of the flagstone floor using a vapour-proof barrier did not increase the moisture levels within the rubble-fill, masonry wall at Court House.
4. The in-situ measurements of wall moisture at Court House contradicted predictions based on a theoretical model of capillary rise for an idealised wall. This is because the heterogeneous fabric of the rubble-fill wall (from visual inspection) seemed to contain a discontinuous pore network between the materials forming the wall. This restricted capillary flow between the wall elements and capillary rise within the wall.

## Data Availability

The datasets generated during and/or analysed during the current study are available in the repository: https://doi.org/10.15125/BATH-01101
